# How Ethical Leadership Prompts Employees’ Voice Behavior? The Roles of Employees’ Affective Commitment and Moral Disengagement

**DOI:** 10.3389/fpsyg.2021.732463

**Published:** 2022-01-20

**Authors:** Jin Cheng, Xin Sun, Jinting Lu, Yuqing He

**Affiliations:** ^1^School of Management, Xiamen University, Xiamen, China; ^2^School of Journalism and Communication, Xiamen University, Xiamen, China

**Keywords:** ethical leadership, voice behavior, moral disengagement, affective commitment, affective events theory (AET)

## Abstract

Previous literature has demonstrated that ethical leadership could predict employees’ voice behavior. However, it’s not clear how to heighten these positive effects of ethical leadership on employees’ voice behavior. Building on the AET and moral disengagement studies, we developed an integrated model. A three-wave field study (*N* = 232) investigated the relationship between ethical leadership and voice behavior by focusing on the mediating role of employees’ affective commitment and the moderating role of employees’ moral disengagement. Our matched data analysis results indicated that: (1) employees’ affective commitment partly mediated the relationship between ethical leadership and employees’ voice behavior. In addition, employees’ moral disengagement moderated (2) the effect of ethical leadership on employees’ affective commitment and (3) the effect of employees’ affective commitment on voice behavior, similarly, (4) the indirect effect of ethical leadership on employees’ voice behavior *via* employees’ affective commitment. Theoretical and practical implications of these results are discussed.

## Introduction

In an increasingly competitive market environment, employee voice — non-required behavior that emphasizes the expression of constructive challenges with an intent to improve rather than criticize — is an excellent way to help organization innovation and maintain sustainable development (LePine and Van Dyne, 1998; Hussain et al., 2019; Xue, 2020). Unfortunately, even when an organization has an open-door policy or anonymous voice mailbox, employees remain silent and do not share their ideas or concerns with the organization (Milliken et al., 2003; Song, 2018b; Hussain et al., 2019).

Scholars have found that many factors can hinder employees from voicing (Kakkar et al., 2016). For example, employees may fear isolation, misunderstanding, retaliation, bias, and low-performance ratings (Detert and Burris, 2007; Detert and Edmondson, 2007). In addition, employees may feel that their voices are not easily heard and adopted (Detert et al., 2010). Therefore, in many cases, employees will remain silent. Many of the structural, interpersonal, and psychological barriers to employees’ vocal behavior may be created and manipulated by their supervisors or leaders (Li and Tangirala, 2021). Therefore, the behavior of leaders is the critical factor affecting employees’ voice behavior, and the relationship between a leader’s behavior and employees’ voice behavior is also the most discussed (Peng and Wei, 2020).

Studies found that ethical leaders pay more attention to employees’ opinions and support their voice behavior (Chen and Hou, 2016; Javed et al., 2018) and make employees feel safe to voice (DeConinck, 2015; Bai et al., 2019). Many studies have also confirmed the positive impact of ethical leadership on employees’ voice behavior (Avey et al., 2012; Lee et al., 2017; Zhou and Wang, 2018; Ng et al., 2021). In particular, scholars have studied the positive role of ethical leadership in promoting employees’ proactive work attitudes and customer-oriented behavior in the retail and healthcare industries (Lindblom et al., 2015; Zappala and Toscano, 2020). In these industries, employees keep close and frequent contact with customers. They are able to identify and grasp the most basic and essential needs of customers, and therefore their voice contains valuable market information. Recently, there has been a growing interest in the mediating mechanisms behind these relationships. The initial proposal of ethical leadership construct and subsequent research on ethical leadership have been based on social learning processes, social exchange processes, and social identity processes (Brown et al., 2005; Zhu et al., 2015), and these explanations have almost always been cut from a rational perspective, leaving research from an affective perspective largely unexplored (Liu et al., 2017).

Despite growing academic interest in the role of affect in work settings, researchers have so far viewed voicing as a calm and rational process (Song, 2018a; Xue, 2020). A typical characteristic of voice behavior is that voicers often decide whether and what to say after weighing the pros and cons and the gains and losses. They are always judging whether their voice is helpful or not, and they do not take risks to speak, nor make voice as they wish. As a result, they have hidden their affect and emotion (Weiss and Cropanzano, 1996; Grant, 2013). This phenomenon has been observed and explained by previous scholars. Nevertheless, this paper attempts to redress this phenomenon by critically reviewing the role of affect, focusing on the influence of affect in the voicing process.

Our research on the role of affect in the relationship between ethical leadership and employees’ voice behavior is based on Affective Event Theory (AET) (Weiss and Cropanzano, 1996). AET emphasizes that the accumulation of a succession of positive or negative events will lead to employees’ positive or negative affective responses, which largely determine the employees’ attitude and behavior (Cropanzano et al., 2017). Consistent with the AET, we propose that perceived ethical leadership is the best example of facilitating the accumulation of positive work events that lead to positive emotional responses that drive employee behavior. Therefore, we investigated the mediating roles of affective commitment between ethical leadership and employees’ voice behavior. In this process, affective commitment is an essential affective factor triggering prosocial activities, which is beneficial to organization development.

Moreover, while previous research has demonstrated some positive effects of ethical leadership, we still know very little about what kind of employee ethical leaders have influence over and what kind of employee is more likely to be motivated to voice. Considering the emphasis of ethical leadership on ethics, employees with different ethical cognitive tendencies will respond differently to ethical leadership and make very different voice behavior decisions (Zhou and Wang, 2018; Moore et al., 2019). At the same time, according to the theoretical framework of AET, employees’ affective response to leaders’ ethical behavior is moderated by individual personality traits (Weiss and Cropanzano, 1996), such as moral disengagement. Therefore, we posit that moral disengagement moderates the relationship between ethical leadership and affective commitment.

As a corollary of this argument, we contend that employees’ moral disengagement also moderates the relationship between affective commitment and voice behavior. Since voice behavior is related to the individual’s tolerance for wrongdoing or unethical behavior in the organization, employees with low moral disengagement are more intolerant of organizational misconduct and more willing to take responsibility and risk for the organization’s growth. Previous studies have suggested that moral disengagement plays an essential role in fostering deviant conduct and hindering prosocial and helping behavior (Baron et al., 2015; Fida et al., 2018; Lian et al., 2020). Our study claims that when employees have low levels of moral disengagement, their affective commitment improves their voice behavior. Conversely, when they have a high degree of moral disengagement, they will lack the courage and responsibility to undertake any form of extra-role behavior.

**FIGURE 1:**
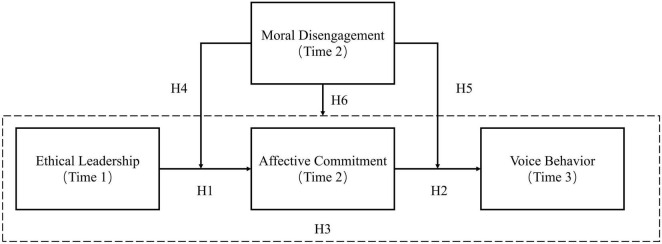
Theoretical Model.

In summary, our research has two objectives. As mentioned above, the first is to investigate the potential of affective commitment as a mediating mechanism between ethical leadership and employees’ voice behavior. The second is to test the moderating effect of moral disengagement on ethical leadership, employees’ affective commitment, and voice behavior. This study has used three-wave data empirically testing research hypotheses in the context of Chinese organizations. The research model is shown in Figure 1.

## Theoretical Background and Hypotheses Development

### Ethical Leadership and Employees’ Voice Behavior

Ethical leadership is defined as “the demonstration of normatively appropriate conduct through personal actions and interpersonal relationships, and the promotion of such conduct to followers through two-way communication, reinforcement, and decision-making” (Brown et al., 2005). If a leader is to have a reputation for being ethical, he/she must be an ethical person and an ethical manager (Trevino et al., 2000; Ahn et al., 2018). As an ethical person, he/she has ethical characteristics such as honesty and integrity (Caldwell et al., 2008). As an ethical manager, he/she adopts ethical strategies that influence organizational ethics and behavior (Peng and Wei, 2020). Some studies on ethical leadership have confirmed that it has a positive effect on subordinate’s performance (Walumbwa et al., 2011), work effort (Haller, 2018), creativity (Feng et al., 2018; Mo et al., 2019), ethical behavior (Moore et al., 2019; Halbusi, 2021), such as prosocial behavior (Piccolo et al., 2010; Kacmar et al., 2011), green behavior (Saleem et al., 2020) and OCB (DeConinck, 2015; Gerpott et al., 2019).

The influence of ethical leadership on employees’ ethical behavior can be explained from different perspectives. From the social learning perspective, ethical leaders are role models and will be imitated by their employees (Bandura and Walters, 1977; Bandura, 1986). Ethical leaders are altruistically motivated, and they are likely to take actions against unethical behavior (Trevino et al., 2000; Brown et al., 2005; Marquardt et al., 2020). As a result, employees will look to ethical leaders as role models. From the social exchange perspective, employees choose their actions primarily based on their relationship with their leader (Walumbwa et al., 2011; Zhou, 2020). Ethical leaders who are caring, fair, and concerned about their employees can earn their trust and loyalty. In turn, employees are willing to reward their leaders by proactively offering constructive ideas and suggestions (Walumbwa and Schaubroeck, 2009). From the social norm perspective, ethical leaders create ethical behavior norms through communications and interactions among colleagues and rewards and punishments within the organization (Keck et al., 2020). Once moral or correct codes of conduct are established, they will promote employees’ ethical behavior just like voice behavior (Avey et al., 2012).

Voicing is “*a discretionary communication of ideas, suggestions, concerns, or opinions about work-related issues with the intent to improve organizational functioning*” (Burris et al., 2008; Detert et al., 2010). As an extra-role behavior, to voice is a bold choice made by employees for the organization’s interests (Morrison, 2011; Lee et al., 2017).

However, voicing is inherently challenging compared to other forms of extra-role behavior, such as helping behavior (Li and Tangirala, 2021). Voicing implies that employees point out flaws in organizational procedures and errors or mistakes of leaders or employees, which may imply irresponsibility on the part of leaders and dereliction of duty on the part of employees. Therefore, it may embarrass leaders or colleagues and damage their interpersonal relationships with vocal employees (LePine and Van Dyne, 1998; Hsiung, 2012; Hussain et al., 2019).

Considering these potentially challenging consequences, the antecedents of voice behavior are complex and involve a delicate balance of consequences in deciding whether or not to vocalize (Li and Tangirala, 2021). Morrison outlined two categories of factors that induce voice behavior: motivators and inhibitors. Motivators are the driving force behind voice behavior, while inhibitors are the restraining forces that pull employees toward silence and reduce their likelihood of speaking. Morrison notes that while many motivators have been empirically confirmed (including ethical leadership), other motivators factors have received less empirical attention, including affect (e.g., affective commitment).

In conclusion, ethical leadership can influence employees’ voice behavior through various mechanisms. However, previous empirical studies on the relationship between ethical leadership and employees’ voice behavior have only emphasized the affective effect of ethical leadership on employees and lacked attention to the affective impact of ethical leadership (Liu et al., 2017). As an affective factor, affective commitment can provide a new mechanism to explain voice behavior according to AET theory (Knoll and Redman, 2016). Affective commitment can lead to persistence during the action, even in the face of conflicting motivations or attitudes. It may even lead individuals to behave in ways that may be contrary to their self-interest from the perspective of a neutral observer (Herscovitch and Meyer, 2002). Thus, it is an essential complement to existing motivational theories and provides a new understanding of voice behavior in the work environment. One of the main objectives of this study is to extend the research on the relationship between ethical leadership and employee voice by introducing a new mediator, affective commitment.

### Establishing a Conceptual Model

This study tries to develop a conceptual model based on affective commitment and Affective Events Theory (AET) literature. Our first theoretical foundation is AET, which is mainly used to explain the relationship between the events experienced by organizational members in the workplace, affective reactions, and the attitudinal and behavioral changes caused by these affective reactions (Weiss and Cropanzano, 1996; Brief and Weiss, 2002). Affective reactions emphasize the individual’s psychological experience and have a multidimensional structure; they manifest as a short-term intense emotional experience or a relatively stable and lasting affective state (Weiss et al., 1999; Beal et al., 2013). This study focuses on this enduring affective state, which can influence an individual’s attitude and behavior toward work and organization (Judge et al., 2017). Our second theoretical foundation is literature on affective commitment.

On the one hand, affective commitment is defined as “*the employees’ affective attachment to, identification with, and involvement in a particular organization, essentially representing the desire to stay in the organization”* (Meyer and Allen, 1984; Meyer et al., 2004). According to Meyer and Allen (1991), affective commitment is a state of affective attachment that employees develop to an organization, a long-term, relatively moderated form of affective response. We believe that the series and accumulation of employees’ experience at work can change the long-term affective response. On the other hand, Herscovitch and Meyer’s extended three-components model indicated that employees’ affective commitment is associated with higher levels of behavioral support for change than other forms of commitment (Herscovitch and Meyer, 2002). Employees’ voice behavior symbolized a high degree of change orientation because voice represented employees’ questioning of the *status quo* and implied a tendency to change the *status quo* to solve problems. Based on this, we choose AET and the literature on affective commitment as the basis for our theory.

#### Ethical Leadership and Affective Commitment

The leader is usually the spokesperson and head of the organization. Therefore, the leader’s behavior will be perceived as organizational behavior, and how employees feel about leaders will translate into how they feel about the organization (Peng and Wei, 2020). According to AET, Cropanzano indicates that leaders can influence subordinates’ affect through daily behaviors and emotional expression, thus influencing the development of their relationship and changing subordinate’s behavior (Cropanzano et al., 2017). We believe that when employees interact with ethical leaders in their daily lives, they experience the accumulation of a series of positive emotional events that form employees’ affective commitment.

There are three reasons to explain our conjecture. Firstly, when leaders are ethical, they treat the employees fairly, concernedly, and responsibly. They care about employees’ interests and provide feedback on their expressed concerns (Brown et al., 2005). It suggests that employees’ trust in these leaders is worthwhile, which in turn enhances employees’ affective organizational commitment (Caldwell et al., 2008; Leroy et al., 2012; Kehoe and Wright, 2013; Devece et al., 2016). Secondly, when leaders are ethical, they prioritize the organization’s sustainability (Eisenbeiss, 2012; Haar et al., 2019). When making decisions, such leaders always ask “what is the right thing to do?” This focus on sustainability and rightness helps ensure the organization’s long-term growth, which increases employees’ confidence in the organization’s future. Thirdly, when the leader is ethical, employees enjoy more job-related freedom, and they show a higher willingness to initiate change (Bormann and Rowold, 2016), enhancing their confidence in the organization.

For these three reasons, ethical leadership fosters positive affective bonds between employees and the organization. Besides, some recent surveys indicate that the correlation coefficient between ethical leadership and affective commitment is significant (Demirtas and Akdogan, 2015; Asif et al., 2019). We predict:

Hypothesis 1: Ethical leadership is positively related to employees’ affective commitment.

#### Affective Commitment and Voice Behavior

To some extent, voicing is challenging because it questions existing problems and breeds organizational change, so many employees hesitate to express their concerns or propose suggestions to improve the organization (Detert and Burris, 2007; Detert and Edmondson, 2007). However, employees with strong affective commitment are more attentive to organizational issues and more motivated to improve (Leroy et al., 2012). In Herscovitch and Meyer’s extended three-component model, employees with higher affective commitment exhibit more supportive behaviors in the face of changes that benefit organizational goals (Herscovitch and Meyer, 2002). Specifically, these supportive behaviors include boldly questioning the organization’s existing problems and making suggestions that will help the organization in the long run, which are primary forms of voice behavior (Chamberlin et al., 2018). They are willing to take the risk of being vocal for the organization’s benefits, regardless of personal gain or loss, and want to contribute to the organization by communicating their ideas (Ng and Feldman, 2012; Song, 2018b; Zhou and Wang, 2018).

On the other hand, it is reasonable to assume that employees with high affective commitment are more willing to stay. Every employee has an intrinsic need to grow and develop (Mignonac et al., 2015; Li et al., 2016). The personal growth of employees is closely related to the development of the organization. Previous studies have shown that positive affect (e.g., positive mood and psychological safety) or attitudes (e.g., organizational identity) are associated with the occurrence of voice (Grant, 2013; Knoll and Redman, 2016; Xue, 2020). Therefore, we propose the following hypothesis:

Hypothesis 2: Affective commitment is positively related to employees’ voice behavior.

Based on the above literature review and discussion, affective commitment may be an essential mediator of ethical leadership in prompting employee voice behavior. Thus, we propose the following hypothesis:

Hypothesis 3: Affective commitment mediates the relationship between ethical leadership and employees’ voice behavior.

### The Moderating Role of Moral Disengagement

Employees’ traits may also influence their organizational commitment. Thus, in light of the influence of ethical leaders on employees’ voice behavior, personal traits of employees, such as moral disengagement, would affect this correlation. This study examines the moderating role of moral disengagement, a key personality trait associated with employees’ perception of ethical and unethical behavior.

Moral disengagement is defined as “*an individual’s propensity to disengage morally — that is, an individual inclination to behave unethically without feeling guilt or distress*” (Bandura, 1999; Newman et al., 2020). According to Bandura’s theory, moral disengagement helps reduce discomfort and self-blame when people act against their ethical standards (Gonzalez, 2018; Newman et al., 2020). Moral disengagement also provides reasons to rationalize unethical and unjust behavior without feeling upset (Fida et al., 2018; Lian et al., 2020). Thus, it plays a crucial role in explaining how individuals can engage in human atrocities such as political and military violence or corporate wrongdoing and corruption without apparent cognitive distress.

Bandura proposed that moral disengagement occurs through three cognitive mechanisms: relabeling unethical or unjust behavior as not unethical, alienating and distorting the harmful consequences of unethical or unjust behavior, and dehumanizing the victims of unethical behavior (Bandura, 1999, 2002). Moral disengagement is a process of rationalizing unethical behavior (Lian et al., 2020). It can also be seen as a personal characteristic that invalidates moral self-regulation and allows individuals to engage in unethical behavior without feeling guilty or uncomfortable (Moore, 2008; Cheng et al., 2019; Schaefer and Bouwmeester, 2020). This research viewed moral disengagement as an unchangeable individual characteristic (Detert et al., 2008). It assumes that moral disengagement moderates the relationship between ethical leadership and affective commitment and the relationship between affective commitment and voice behavior.

#### Moral Disengagement’s Moderating Role in the Relationship Between Ethical Leadership and Affective Commitment

Individuals with low moral disengagement maintain high ethical standards and are more likely to feel guilty about their own and others’ unethical behavior (Bandura, 1999; Sherman, 2020). According to the interpersonal attraction theory, people often consciously or unconsciously approach people with similar characteristics to establish trust and mutual identification (Paciello et al., 2008; Moore, 2016). Therefore, employees with low levels of moral disengagement are more sensitive to the ethical behavior of ethical leaders and identify more with their leaders (Gonzalez, 2018). This identification and respect are projected into the organization, increasing employees’ affective commitment.

On the contrary, those with a high tendency toward moral disengagement are free from self-sanction and attendant guilt when engaging in unethical behavior (Detert et al., 2010; Gonzalez, 2018). These people dilute the moral implications of unethical behavior by rationalizing it. They can easily overlook the ethical behaviors of leaders and do not fully understand the value of these ethical behaviors (Paciello et al., 2008). As a result, they do not particularly respect and appreciate ethical leadership and produce a range of positive affective responses. Therefore, the effects of ethical leadership on affective commitment will be weakened. Previous research has shown that when leaders and followers share similar values, it may also enhance employees’ psychological attachment to the organization (Hogg and Turner, 1985). Based on the above discussion, we propose:

Hypothesis 4: Moral disengagement moderates the relationship between ethical leadership and affective commitment; the positive relationship between them will be weakened under conditions of high moral disengagement.

#### Moral Disengagement’s Moderating Role in the Relationship Between Affective Commitment and Voice Behavior

Some scholars have confirmed that people with a high degree of moral disengagement are likely to judge unethical behavior as ethical and make an unethical decision (Bandura, 2002). A highly morally disengaged person will believe that his/her unethical or unjust behavior is justified or beneficial within the organization and may blame others for wrongdoing (Lian et al., 2020). Due to the inertia of individual thinking, it is easy for them to use the same thinking to understand misconduct in the organization (Gonzalez, 2018). As a result, individuals may ignore or rationalize negative organizational information about misconduct or even view it as a functional aspect of the organization. In this case, his/her affective commitment reduces the likelihood of voicing to protect the company’s image. Moreover, a highly morally disengaged person will lack the moral courage to challenge the organization’s *status quo* and tend to shift responsibilities to others, despite his/her affective commitment to the organization.

Conversely, if a person is highly morally engaged, he/she will be more enthusiastic about identifying unethical behavior and will not tolerate unethical behavior in the organization. At the same time, given the challenging nature of voicing, a highly morally engaged person will take responsibility to voice their concerns or suggestions to the organization and transfer his/her great affection toward the organization into practical action — voice. Given the above discussion, we suggest that:

Hypothesis 5: Moral disengagement moderates the relationships between affective commitment and voice behavior; the positive relationship between them will be weakened under conditions of high moral disengagement.

#### Moral Disengagement’s Moderating Role in the Indirect Effect of Ethical Leadership on Employees’ Voice Behavior *via* Employees’ Affective Commitment

Combining Hypotheses 4 and 5, we predict that moral discouragement can weaken the indirect relationship between ethical leadership and voice *via* affective commitment. We also propose a moderated mediation relationship:

Hypothesis 6: Moral disengagement moderates the mediating effect of ethical leadership on voice behavior *via* affective commitment. The indirect relationship will be weaker when moral disengagement is higher.

## Methodology

### Data Collection and Procedures

This study adopted a cross-sectional design with an individual level of measurement and analysis. Data were collected from only one organizational hierarchy. Specifically, we collected data from 15 retailing companies located along the southeastern coast of China. Retail is particularly close to people’s daily lives, and retailers need to gather their employees’ voices on customer feedback, product quality control, etc. Previous sound studies have collected samples from a single industry (e.g., Walumbwa and Schaubroeck’s research into a large financial institution) (Walumbwa and Schaubroeck, 2009) because this focus has a substantial advantage, that is, “unknown sources of variance due to the organization type can be controlled” (Near et al., 2004). We chose the retail industry as our research sample, following the same logic. The specific sampling process was as follows:

In terms of random sampling, we took a series of measures to ensure the randomness of the sample. As previous scholars asked for help from directors or supervisors (see Kacmar et al., 2013), our sampling process was also conducted with the help of HR. Firstly, we set a series of principles of random sampling designed for this study, and then asked HR to select 20 ∼ 60 employees randomly from different departments of each company according to this random sampling method; after that, we got a total of 600 employees as the sample, and we randomly assigned a three-digit code to each employee. Secondly, before filling in the formal questionnaire, researchers gave detailed instructions and explanations for employees, emphasizing the anonymity and confidentiality of the whole data collection process, and repeatedly stressed that participation was voluntary, so the voluntariness of the respondents also ensured the randomness of sampling; Thirdly, HR issued questionnaires to those selected employees (as some employees are away from the company on business or for other reasons, the number of questionnaires issued each time is about 540).

As mentioned before, this study adopted a three-wave data collection method to reduce the common method variance (CMV) (Podsakoff et al., 2003). Hansen et al. (2013), Liao et al. (2015) and Lee et al. (2017) chose a 1-month interval when studying the influence of ethical leadership to fully observe the impact of ethical leadership on outcome variables while reducing CMV. We also adopted a 1-month interval in the data collection process following their recommendation.

At Time 1, each envelope was marked with a three-digit number and distributed to employees. Envelopes were handed out to 543 employees because some did not show up for work for business reasons. We then received a total of 282 responses, including respondents’ demographic information (e.g., age, gender, education, position), department size, and items about ethical leadership. One month later, at Time 2, we collected the data on affective commitment and moral disengagement, and this time we received 278 responses. At Time 3, 1 month after Time 2, we collected the questionnaires focusing on voice behavior and got 275 responses in total. Finally, 239 matching questionnaires were identified by matching the three-wave questionnaires with three-digit numbers. We then omitted seven invalid questionnaires (where over 5% of the data were missing), leaving 232 valid samples.

Regarding sample size adequacy, Bentler and Chou (1987) suggested that the appropriate sample size is about 5 to 10 times the number of items to run the SEM. Generally, *N* = 100–150 is contemplated as the least possible sample size for conducting SEM (Ding et al., 1995; Kyriazos, 2018). Besides, we also calculated the sample size using software, the first one was G*Power 3.1, α = 0.05, power = 0.95, *f*^2^ = 0.15, the calculated sample size was 89 cases; the second one was PASS15, α = 0.05, power = 0.95, *f*^2^ = 0.15 (the medium effect size), the calculated sample size was 97 cases.

To sum up, the sample size used in this study (*N* = 232) was much larger than those calculated by the software, and at the same time fulfilled standards recommended by previous scholars (Bentler and Chou, 1987; Kock and Hadaya, 2016; Kyriazos, 2018), then the sample size of this study seemed adequate and justified to run the SEM for data analysis, fulfilling the minimum sample size requirement.

Table 1 provides the sample characteristics, 40.5% were male, 59.5% were female, 41.4% were 31–40 years old, and 69.0% had a bachelor’s degree, 58.2% of the sample held low-level positions. As for tenure, 35.3% had been in their jobs for more than 8 years. Only 0.9% of the samples belonged to the R&D department, while 50.9% belonged to the management department. Finally, nearly half have more than 20 employees.

**TABLE 1:** Sample characteristics (*n* = 232).

Category	Characteristics	*n*	%
Gender	Male	94	40.5
	Female	138	59.5
Age	≤ 25	20	8.6
	26–30	70	30.2
	31–40	96	41.4
	>41	46	19.8
Edu	High/Primary school	7	3.0
	Junior college	55	23.7
	Bachelor’s degree	160	69.0
	Postgraduate/doctoral level	10	4.3
Position	Low level	135	58.2
	First–line manager	78	33.6
	Middle manager	19	8.2
Tenure	≤ 1	18	7.8
	1–3	67	28.9
	4–7	65	28.0
	>8	82	35.3
Department	R&D	2	0.9
	Management	118	50.9
	Production	39	16.8
	Sales	5	2.2
	Finance	15	6.5
	Logistics	25	10.8
	Other	28	12.1
Size	≤ 5	11	4.7
	6–10	37	15.9
	11–15	53	22.8
	16–20	20	8.6
	≥ 21	111	47.8

### Variable Measurement

Since all the multi-item measures in this study were initially constructed in English, we developed Chinese versions for all the measures following the commonly used back-to-back translation procedure. After that, we pre-tested four key variables and assessed all of these items on a 5-point-Likert scale ranging from 1 (strongly disagree) to 5 (strongly agree).

#### Ethical Leadership

The 10-item ethical leadership scale was used in the study (Brown et al., 2005), which has been used by many scholars (Avey et al., 2012; Zhu et al., 2015). Example items are: “My supervisor listens to what employees have to say,” “My supervisor sets an example of how to do things the right way in terms of ethics” and “My supervisor disciplines employees who violate ethical standards.” The Cronbach’s alpha for this scale was 0.93.

#### Affective Commitment

Six items from Allen and Meyer were used to measure affective commitment (Allen and Meyer, 1996), including “I would be thrilled to spend the rest of my career with this organization,” “I enjoy discussing my organization with people outside it” and “I feel as if this organization’s problems are my own.” The Cronbach’s alpha for this scale was 0.96.

#### Voice Behavior

A 10-item questionnaire developed by Liang was used to measure voice behavior (Liang et al., 2012). Items of promotive voice behavior include “I proactively develop and make suggestions for issues that may influence the unit,” “I proactively suggest new projects which are beneficial to the work unit,” “I speak up honestly about problems that might cause serious loss to the work unit, even when/though dissenting opinions exist.” The Cronbach’s alpha for this scale was 0.94.

#### Moral Disengagement

Including 24 items, this construct was assessed with a measure similar to the one developed and used in multiple studies by Bandura and others (Bandura, 2002; Pelton et al., 2004). After conducting a pilot study, we retained 19 items. Sample items include “It is okay to spread rumors to defend those you care about,” “People should not be held accountable for doing questionable things when they were just doing what an authority figure told them to do,” “Compared to other illegal things people do, taking something small from a store without paying for it is not worth worrying about.” The Cronbach’s alpha for this scale was 0.92.

#### Control Variables

We controlled for age, sex, education level, position, tenure, and sector size due to the potential effects of individual demographics. Age was coded as “1” representing below 25 years old, “2” representing 26 to 30 years old, “3” representing 31 to 40 years old, and “4” representing over 41 years old. Gender was coded as “1” representing male and “2” representing female. Other variables, like education level, position, tenure, department, and department size, were coded the same way.

### Data Analysis

Although the concept (leadership) seems to be multileveled by nature, researchers are interested in individuals’ perceptions of ethical leadership in the current study (Cheng et al., 2019). We examine the measurement and structural model simultaneously with Partial Least Squares Structural Equation Modeling (PLS-SEM) using SmartPLS 3.0 (Ringle et al., 2015). PLS-SEM enables users to create a single theoretical model and simultaneously examine the direct, indirect, and moderating effects of ethical leadership on affective commitment and voice behavior. A confirmatory composite analysis is the appropriate two-step method for PLS-SEM analysis (Hair et al., 2020). The first step is an evaluation of the measurement model, and the second step is an evaluation of the structural model (Sarstedt and Cheah, 2019). Below we detail the data analysis and results of this study.

### Measurement Model

The descriptive statistics, including means, standard deviations, and correlations for all the variables included in the conceptual model, can be found in Table 2. These results indicate multiple significant relationships between constructs in the structural model, and many instances are consistent with previous findings in the literature. The results also support the reliability and validity of the theoretical measurement models.

**TABLE 2:** Means, standard deviations, and correlation coefficients.

	1	2	3	4	5	6	7	8	9	10
1 Gender	−									
2 Age	−0.129*	−								
3 Education	0.017	−0.240**	−							
4 Position	−0.246**	0.390**	0.098	−						
5 Tenure	–0.095	0.537**	−0.194**	0.307**	−					
6 Department size	–0.046	−0.155*	0.139*	–0.054	−0.136*	−				
7 EL	−0.137*	–0.074	0.190**	0.114	−0.169*	0.008	(0.93)			
8 MD	0.002	–0.093	0.02	–0.043	–0.03	0.018	−0.230**	(0.92)		
9 AC	−0.163*	0.034	0.05	0.095	–0.063	0.07	0.429**	−0.442**	(0.96)	
10 VB	–0.121	0.195**	0.082	0.150*	0.103	–0.022	0.295**	−0.191**	0.393**	(0.94)
Mean	1.59	2.72	2.75	1.5	2.91	3.79	3.96	1.95	3.8	3.9
Standard Deviations	0.49	0.88	0.58	0.65	0.97	1.32	0.78	0.57	0.84	0.64

*N = 232.*

Values in parentheses along the diagonal are Cronbach’s alphas. Gender: 0 = “male” 1 = “female”; Education: 1 = “high school and below high school” 2 = “college” 3 = “Bachelor degree” 4 = “Master degree and above master” Position: 1 = “general staff” 2 = “first-line manager” 3 = “middle manager” 4 = “top manager”; EL, Ethical leadership; AC, Affective commitment; VB, Voice behavior; MD, Morel disengagement; **p < 0.01; *p < 0.05.

Measurement model assessment begins with evaluation and confirmation of reliability and validity of the outer measurement models. Results of the initial measurement model assessments are shown in Table 3. All indicators exhibited acceptable item reliabilities. All measurement models (constructs) were above the recommended levels of.70 for composite reliability. Overall, all requirements for measurement model reliability were well above recommended minimum guidelines (Hair et al., 2021). The convergent validity of the measurement model can be assessed by the Average Variance Extracted (AVE). The AVEs of most constructs were well above the minimum recommended level of.50 (Hair et al., 2021), while the AVE of Moral disengagement was 0.408, which was not qualified. Since Henseler et al. (2015) proposed that Fornell and Larcker’s (1981) criterion and cross-loadings were not sufficient for discriminant validity, we then assessed the discriminant validity between constructs using the heterotrait-monotrait (HTMT) method. The HTMT values were between 0.194–0.468, less than the strict standard of 0.85 (Kline, 2015). Thus, discriminant validity among all constructs was confirmed.

**TABLE 3:** PLS-SEM: Reliability, Validity, and AVEs.

	CR	1	2	3	4
1. EL	0.945	**0.636**			
2. AC	0.937	0.460	**0.713**		
3. VB	0.948	0.311	0.424	**0.648**	
4. MD	0.929	0.249	0.468	0.194	**0.408**

*EL, Ethical leadership; AC, Affective commitment; VB, Voice behavior; MD, Morel disengagement; CR represents composite reliability; The diagonal in bold is the square root of the average variance extracted (AVE), other numbers along the diagonal are HTMT (heterotrait-monotrait).*

### Structural Model

The second step of the confirmatory composite analysis process is the structural model’s assessment (Hair et al., 2020). We used PLS Algorithm to analyze the structural model. Standardized Root Mean Square Residual (SRMR) could be used to evaluate model fitness. When the SRMR of the Saturated Model and the Estimated Model is less than 0.08, they have a good fit (Hu and Bentler, 1999), and the smaller the gap between the values of the Saturated Model and the Estimated Model, the better (Henseler et al., 2015). The results of this structural model showed that the values of the saturation model and estimation model were 0.061 and 0.063, respectively, so the model fit well.

**FIGURE 2:**
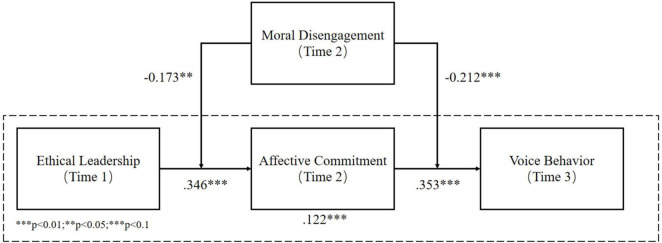
Structural model with parameter estimates (SmartPLS3).

Then, we examined the path coefficients and significance levels for the hypothesized relationships. These metrics were obtained by executing the PLS bootstrapping procedure. We used 5,000 samples to produce bias-corrected confidence intervals for each coefficient for this procedure. The hypothesized direct relationships were examined first, and then the hypothesized indirect relationships. Figure 2 provides an overview of the results.

#### Direct Relationships

Figure 2 shows the estimation results. Hypothesis 1 proposes a positive relationship between ethical leadership and affective commitment, and Hypothesis 2 proposes a positive relationship between affective commitment and voice behavior. The results indicate that both of these relationships are significant. Thus, H1 and H2 were accepted; the results are shown in Table 4.

**TABLE 4:** PLS-SEM Direct Relationships: Standardized Path coefficients and results of Hypothesis.

	Original sample	Accept/reject and significance	Hypothesis
EL→AC	0.346	Accept (0.000)***	H1
AC→VB	0.353	Accept (0.000)***	H2

*EL, Ethical leadership; AC, Affective commitment; VB, Voice behavior; MD, Morel disengagement.*

***p < 0.001.

#### Indirect Relationships

As for indirect relationships, mediation occurs when a mediating variable is placed between exogenous and endogenous related constructs. Progressively, when there is a change in the exogenous variable, it changes the mediator variable, which subsequently impacts the endogenous variable (Hair et al., 2021). We now report the results for the indirect mediating relationships.

Hypotheses 3 proposes that affective commitment mediates the relationship between ethical leadership and employees’ voice behavior. Results show that the positive relationship between ethical leadership and voice behavior through affective commitment was significant. Thus, Hypothesis 3 is supported (Table 5).

**TABLE 5:** PLS-SEM Indirect Relationships (Mediation): Standardized Path Coefficients and Results of Hypothesis.

	Original sample	Accept/reject and significance	Hypothesis
EL→AC→VB	0.122	Accept (0.000)***	H3

*EL, Ethical leadership; AC, Affective commitment; VB, Voice behavior; MD, Morel disengagement.*

***p < 0.001.

**FIGURE 3:**
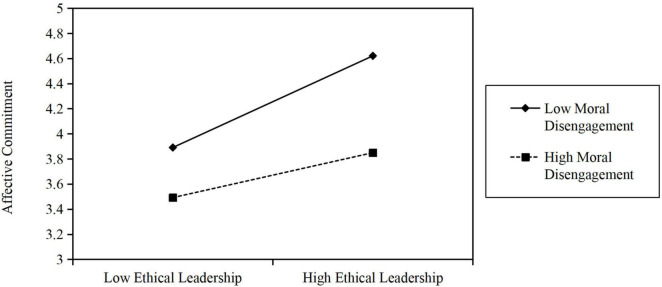
Simple Slope Moderating Analyses: Significant moderation of MD between EL and AC. EL, Ethical leadership; AC, Affective commitment; VB, Voice behavior; MD, moral disengagement.

Next, the moderating process is dependent on moral disengagement in this study (including Hypothesis 4, Hypothesis 5, and Hypothesis 6). In Hypothesis 4, we hypothesize that moral disengagement will moderate the relationship between ethical leadership and affective commitment, and the relationship is stronger when moral disengagement is low. The interactive effect of moral disengagement and ethical leadership on affective commitment was significant (β = −0.173, *t* = 2.339, *p* = 0.019). In addition, simple slope analysis showed that the slope of ethical leadership on affective commitment with 95% confidence intervals for the indirect effect excluded zero (CI_95%_: −0.327, −0.123) (see Figure 3). Since moderation describes a change in strength and/or direction in the relationship between two constructs that can be impacted by a third moderator construct. Thus, Hypothesis 4 is supported.

**FIGURE 4:**
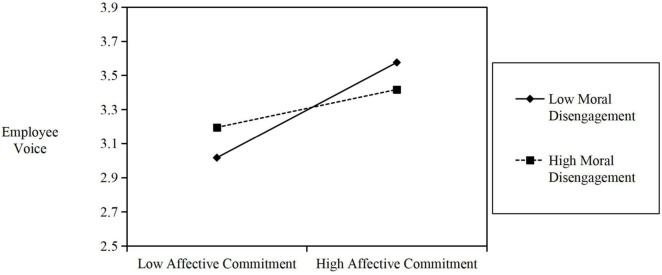
Simple Slope Moderating Analyses: Significant moderation of MD between AC and VB. EL, Ethical leadership; AC, Affective commitment; VB, Voice behavior; MD, moral disengagement.

Hypothesis 5 predicts that moral disengagement will moderate the relationship between affective commitment and voice behavior, i.e., the relationship is stronger when moral disengagement is low versus high, the interactive effect of affective commitment and moral disengagement on voice behavior was also significant (β = −0.212, *t* = 2.664, *p* = 0.008). In addition, the slope of employee voice on affective commitment was significant, with 95% confidence intervals for the indirect effect excluded zero (CI_95%_: −0.379, −0.171) (see Figure 4). Thus, Hypothesis 5 is supported.

Hypothesis 6 predicts that moral disengagement moderates the indirect positive effect of ethical leadership on voice behavior. Results show that ethical leadership’s indirect effect on voice *via* affective commitment was significantly moderated by moral disengagement (β = −0.061, *t* = 2.103, *p* = 0.035). In supporting this effect, bootstrapping analyses demonstrated that 95% confidence intervals for the indirect effect excluded zero (CI_95%_: −0.124, −0.028). Therefore, moral disengagement negatively moderates the effect of ethical leadership on voice *via* affective commitment (see Table 6). Thus, Hypothesis 6 is fully supported.

**TABLE 6:** PLS-SEM Indirect Relationships (Moderation): Standardized Path Coefficients and results of Hypothesis.

	Original sample	Accept/reject and significance	Hypothesis
EL*MD→AC	−0.173	Accept (0.019)**	H4
AC*MD→VB	−0.212	Accept (0.008)***	H5
EL*MD→AC→VB	−0.061	Accept (0.035)**	H6

*EL, Ethical leadership; AC, Affective commitment; VB, Voice behavior; MD-Morel disengagement.*

***p < 0.001; **p < 0.01; *p < 0.05.

## Discussion

### Theoretical Implications

Our findings contribute to the literature on ethical leadership and voice behavior. First, this study extends the research on ethical leadership by adding a substantive mediator to explain the influence of ethical leaders on employee voice behavior. Second, our study provides a new perspective that employees’ affective commitment to the organization is a powerful motivation for employees to speak up when motivated by ethical leadership. When employees have this kind of attachment to the organization, they are willing to accept the risks involved (e.g., extra work and cost) and challenge the *status quo* by expressing their opinions. Although some previous research has described how ethical leadership promotes employee voice behavior, most focus on rational mechanisms. Employees choose whether or how to voice based on the benefits and costs of voice behavior and the success or failure of voice behavior. The existing mediators, such as psychological safety (Walumbwa and Schaubroeck, 2009; Zhou and Wang, 2018), organizational ethical culture and climate (Demirtas and Akdogan, 2015; Bai et al., 2019), unethical climate (e.g., political climate) (Cheng et al., 2019), and individuals’ moral efficacy (Lee et al., 2017), are all understood from the rational perspective. These mediators seldom consider the complex role of leadership on outcomes. Therefore, based on AET, this study proposes that affective commitment to the organization serves as the mediating mechanism by which ethical leadership stimulates employees’ voices behavior, enriching the understanding of the relationship between ethical leadership and unique extra-role behavior.

Second, our findings suggest that the crucial role of employees’ moral disengagement as a condition that strengthens or weakens the expected response to perceived ethical leadership. Previous research has not considered the personal traits of subordinates, which may be related to the leader’s influence and the degree to which the leader assimilates them. Employees with a low degree of moral disengagement are more likely to experience psychological comfort or empathy when observing or perceiving the ethical behavior of leaders. Therefore, for employees with a low degree of moral disengagement, leaders’ ethical behavior has a more significant impact on their affective commitment to the organization. On the contrary, when the moral disengagement degree of employees is high, it is difficult for ethical leadership to promote their affective commitment to the organization.

Third, our study enriches proactive behavior theory (including voice). Many previous studies believe that organizational commitment promotes employees’ proactive behavior (Lapointe and Vandenberghe, 2018), while others do not. For example, Dean and Greene (2017) suggest that attachment to an organization blocks voice (Dean and Greene, 2017). In research by Parker et al. (2006), empirical evidence also indicates that affective organizational commitment has nothing to do with proactive behavior (an integrating measure including proactive idea implementation and proactive problem solving). Our results show that the shift from affective commitment to extra-role behavior such as voice depends on individual traits, especially moral disengagement. While affective commitment is associated with an employees’ willingness to contribute to the organization, it does not necessarily bring attention to issues or motivate them to take action, especially by voice. Voice behavior is closely related to an individual’s judgment of right and wrong and internal moral standards. It involves the assessment of problems existing in the organization and the willingness of employees to take responsibility for their suggestions. Therefore, an individual’s moral disengagement can explain what conditions affective commitment will bring voice behavior. Compared with just saying (e.g., voice behavior), doing (e.g., proactive behavior) needs to overcome more difficulties and require the actor to have a higher moral standard. Further research should consider moral disengagement as a moderator to explain the relationship between the attachment to the organization and more holistic, proactive behavior.

Finally, the overall integrated moderated mediation model provides substantial evidence that the extent to which affective commitment mediates the relationship between ethical leadership and voice depends on the degree of an individual’s moral disengagement. Although previous studies have confirmed that organizational ethical environment or personal factors are related to the relationship between ethical leadership and voice (Zhu et al., 2015; Bai et al., 2019), they remain silent about the conditions under which the mediating effect of situational or personal factors is amplified or attenuated. By identifying this boundary condition, this study contributes to a more accurate understanding of the role of ethical leadership in organizations. Also, it helps explain “the complex ethical leadership-employee performance relationship” (Walumbwa and Schaubroeck, 2009).

### Practical Implications

Our findings may have the following contributions which can be used in organizations in the future. First, our study proved the positive effect of ethical leadership on employees’ psychology and behavior. Many organizations choose managers more based on their competence and performance in the modern business society. However, they do not pay enough attention to the ethical or moral nature of the manager. Our research demonstrates that ethical leadership encourages employees to speak up, which is vital for business development and increasingly important in the VUCA (volatility, uncertainty, complexity, and ambiguity) environment (Detert and Burris, 2007). In a VUCA environment, it is increasingly difficult for its leadership to grasp market trends and consumer behavior trends. To catch these market changes, organizations need to rely on all employees’ knowledge, wisdom, and information and their advice, so ethical leadership has a more critical role in VUCA environments (Schoemaker et al., 2018). Therefore, when selecting and promoting managers, companies should check leaders’ morals and ethics.

Second, our findings indicate that employees’ affective commitment to the organization promotes employees’ voice behavior. In the modern business world, the relationship between companies and employees is very fragile, and companies do not pay much attention to cultivating feelings with employees (Eisenberger et al., 2002). Companies and employees have more transactional relationships than commitment-based relationships. When companies face strategic transformation and business failure, they will ruthlessly lay off employees so that employees will not do their best for the company and give the advice to contribute to its development (Hom et al., 2017). Therefore, companies should care about their employees, respect them, provide competitive salaries and opportunities to enhance their employees’ commitment to the organization.

Third, our results indicate that moral disengagement is vital in promoting employees’ voice behavior in ethical leadership’s function. Employees with a high sense of moral disengagement are unresponsive to ethical leadership and organizational problems. They are reluctant to contribute to the development of the organization. Therefore, organizations should also pay attention to the moral tendencies of their employees when selecting them. They should not choose employees who can easily justify their misconducts; instead, they should select employees who can frequently reflect on themselves and correct their wrongdoing carefully. At the same time, the organization should continually communicate with employees about ethics to increase their sensitivity to ethical issues and guide them to distinguish right from wrong in organizational phenomena more accurately.

### Limitations and Directions for Future Research

Despite these contributions, our research has some limitations. First, data analysis was based on a sample drawn from the Chinese retail industry, limiting our findings’ generalizability. Therefore, future research can replicate this study in other countries with different cultures or contexts to examine and enhance the validity and generalizability.

Second, this study collected data from a single set of respondents. We used perceptual data of the respondents. Although objective and subjective evaluations are not the same, perception measurement is generally an accepted way to measure leadership and employee behavior (Lee et al., 2017; Cheng et al., 2019). Although we collected data in three phases and used statistical tools to examine common method bias, we also used the respondents’ anonymity to ensure the social desirability bias was minimized; we could not completely rule out the possibility of this form of bias. Using alternative measurement methods (e.g., using data from multiple sources and examining both other- and self-reported sources) may help reduce potential perceptual bias in ratings. Future research could use a multilevel approach to analyze the effects of ethical leadership.

Third, regarding the control variables at different times (e.g., control for t1 and t3 when using a variable measured at t2), we used the same control variables to seek uniformity, making it difficult to measure the effects of different control on specific variables. Different control variables should be used in future studies to control their influence on the outcome variables better.

Fourth, while we did examine an integrated theory-related moderated mediation model, other mechanisms could help explain the relationship between ethical leadership and employee voice. Future research should examine different mediating factors such as moral identity in more detail and explore the moderating effects of other personality traits such as emotional intelligence, political skills, situation-specific leverage, the locus of control.

## Conclusion

The relationships between ethical leadership and voice behavior have been widely discussed, but most studies emphasize their rational aspect. Surprisingly, little research has considered these effects from an affective perspective. Our AET-based study found that employees’ affective commitment played a mediating role in the relationship between ethical leadership and voice behavior, in addition, while moral disengagement played a moderating role. Specifically, when employees perceive ethical leadership, low-level moral disengagement individuals will produce more affective commitment and more positive voice behavior than high-level moral disengagement individuals.

## Data Availability Statement

The original contributions presented in the study are included in the article, further inquiries can be directed to the corresponding author.

## Ethics Statement

The study was approved by the Ethics Committee of Xiamen University. Written informed consent from the participants was not required to participate in this study in accordance with the national legislation and the institutional requirements.

## Author Contributions

JC and XS developed the research idea, design, and methodology. XS and YH conducted data collection and revised the manuscript. JL conducted the data analysis, revised the manuscript, interacted with reviewers and editor. YH conducted data collection and prepared the original draft. All authors contributed to the article and approved the submitted version.

## Conflict of Interest

The authors declare that the research was conducted in the absence of any commercial or financial relationships that could be construed as a potential conflict of interest.

## Publisher’s Note

All claims expressed in this article are solely those of the authors and do not necessarily represent those of their affiliated organizations, or those of the publisher, the editors and the reviewers. Any product that may be evaluated in this article, or claim that may be made by its manufacturer, is not guaranteed or endorsed by the publisher.
